# Predictors of early treatment response to antihistamines and omalizumab in chronic spontaneous urticaria

**DOI:** 10.3389/falgy.2025.1728559

**Published:** 2026-01-12

**Authors:** P. Calzari, E. M. Favale, M. Cugno, R. Asero, A. V. Marzano, S. M. Ferrucci

**Affiliations:** 1 Unit of Dermatology, Fondazione IRCCS Ca’ Granda Ospedale Maggiore Policlinico, Milan, Italy; 2 Institute of Dermatology, Fondazione IRCCS Policlinico San Matteo, Pavia, Italy; 3 Department of Pathophysiology and Transplantation, Università Degli Studi di Milano, Milan, Italy; 4 Internal Medicine, Fondazione IRCCS Ca’ Granda Ospedale Maggiore Policlinico, Milan, Italy; 5 Clinica San Carlo, Ambulatorio di Allergologia, Paderno Dugnano, Italy

**Keywords:** biomarkers, chronic spontaneous urticaria (CSU), precision medicine, predictors, therapeutic response

## Abstract

Chronic spontaneous urticaria (CSU) is a common immune-mediated skin disorder characterized by spontaneous wheals, angioedema, or both, persisting for more than six weeks. Its pathogenesis is multifactorial, involving mast cell and basophil activation, autoimmunity and dysregulation of inflammatory and coagulation pathway. Current treatment guidelines recommended a stepwise algorithm beginning with second-generation H1-antihistamines (sgAH1) at standard doses (which can be increased up to fourfold if needed) before progressing to omalizumab (OMA). Nevertheless, a considerable proportion of patients remain unresponsive, highlighting the need for reliable predictors of treatment response to enable personalized care. This narrative review summarizes the current evidence on demographic, clinical, serological, and cellular biomarkers that may predict outcomes with sgAH1and OMA. Favorable sgAH1 response has been associated with shorter disease duration, low baseline UAS7 scores, and absence of angioedema. In contrast, high disease activity, inducible urticaria, elevated CRP or IL-6 levels, and hematological features such as increased neutrophil-to-lymphocyte ratio, basopenia, eosinopenia, and markers of coagulation activation (e.g., D-dimer, fibrinogen) are linked to resistance. Regarding OMA, predictors of good response include high total IgE levels, elevated basophil FcεRI expression, and reduction in IL-31 and D-dimer during treatment. Poor response correlates with advanced age, high BMI, comorbid autoimmune diseases, low total IgE (<40–50 IU/ml), positivity for ANA or anti-TPO antibodies, and activation markers such as CD203c. Functional test like the autologous serum skin test (ASST), basophil activation test (BAT), and histamine release assays offer additional stratification value. Composite immunological signatures integrating multiple biomarkers hold promise for guiding therapeutic decisions and improving prediction accuracy. Implementing validated markers could enable earlier identification of difficult-to-treat patients, faster disease control and more targeted therapy, advancing precision medicine in CSU.

## 1 Introduction

Chronic Spontaneous Urticaria (CSU) is a chronic relapsing-remitting condition characterized by the spontaneous and unpredictable appearance of short-lived wheals, angioedema or both for more than 6 weeks (1). It is a widespread disorder, affecting 0.6%–1.1% of the general population with a higher prevalence in females (2:1 ratio) (2, 3) with uniform distribution across national borders (4) Although it can occur at any age, the peak incidence is observed between 20 and 40 years (5). The diagnosis of CSU remains exclusively clinical. Most patients (57%) have only wheals, 37% have both wheals and angioedema, and 6% have only angioedema (6). It occurs in combination with inducible urticaria (CIndU) in 10%–50% of patients (5). Among the CIndU types, skin writing (dermographism) and cholinergic urticaria are the most common (7). CSU is a self-limited disease; the duration of the disease is generally 1–5 years, but it may persist longer in 10%–14% of cases (5). Pruritus is the hallmark symptom affecting patients' quality of life. It contributes to sleep disturbances, reduced physical and emotional well-being, and decreased performance in both academic and professional environments (8, 9). Furthermore, the psychological sphere is also affected, with an increased rate of anxiety, depression, and somatoform disorders compared to general population (5). This underscores the profound impact of CSU on both individuals and society, especially since it predominantly affects the working-age population (9). The pathogenesis of CSU is multifactorial, involving complex interactions between the immune system, inflammation, and the coagulation pathway (10). Mast cells (MCs) and basophils are the principal effector cells, releasing histamine, cytokines, and other proinflammatory mediators that drive dermal inflammation (11). Two distinct antibody-mediated mechanisms of mast cell activation have been identified: Type I autoallergy (TIaiCSU), in which IgE antibodies are directed against self-antigens and Type IIb autoimmunity (TIIbaiCSU), characterized by the presence of IgG autoantibodies targeting either the *α*-chain of FcεRI or FcεRI-bound IgE. Both pathways may coexist in the same patient. Furthermore, B cell receptor (BCR) contributes to the autoantibody production (12). Interactions between coagulation and complement systems contribute to the underlying pathophysiological mechanisms. Activation of the extrinsic coagulation pathway, triggered by *tissue factor* (TF) expressed by endothelial cells and eosinophils, leads to the generation of thrombin (FIIa) and prothrombin fragment 1 + 2 (F1 + 2). These mediators promote mast cell degranulation via PAR-1 and PAR-2 receptors and contribute to increased vascular permeability and inflammation. Fibrin formation and subsequent fibrinolysis result in the generation of D-dimers. Elevated levels of D-dimer and prothrombin fragment 1 + 2 (F1 + 2) have been consistently detected in severely affected CSU patients, indicating systemic activation of coagulation (10). TF exposition promotes extrinsic coagulation pathway activation amplifying thrombin generation and producing anaphylatoxins (such as C3a and C5a). Furthermore, the coagulation and complement systems are interconnected in the pathogenesis of CSU (13, 14). Chronic tissue damage can trigger neurogenic inflammation, leading to the production of a neuropeptide, substance P (SP). SP, along with eosinophil-derived factors binds to Mas-related G-protein coupled receptor X2 (MRGPRX2) expressed on skin mast cells, stimulating degranulation (15, 16). MRGPRX2 plays a key role in mast cell activation and degranulation through IgE-independent pathways. It can be stimulated by a wide range of endogenous peptide agonists, such as neuropeptides, eosinophil granule proteins, and antimicrobial peptides, as well as by various exogenous compounds, including fluoroquinolone antibiotics, phenothiazines, neuromuscular blocking agents, hormone receptor modulators, iodinate contrast media and certain natural products (17). This pathway may explain why some CSU cases are refractory to anti-IgE therapies (omalizumab - OMA), since MRGPRX2-driven mast cell activation bypasses IgE and FcεRI signaling, rendering IgE-targeted therapies less effective. The current international guidelines recommend a three-step treatment approach (1). The first step is represented by the administration of second-generation H1-antihistamines (SgAHs) at the standard dose. In case of persistent symptoms, the dose may be increased up to fourfold the standard level. However, standard-dose SgAHs provide symptom relief in fewer than 50% of patients. Although higher doses of sgAHs improve treatment outcomes, approximately one in three to four patients remains symptomatic. In such cases, third-line treatment involves the addition of omalizumab. While most patients benefit from this stepwise approach, a subset still fails to achieve adequate symptom control. For these individuals, off-label use of immunosuppressive agents is recommended, with cyclosporine representing the most extensively studied and commonly used option. Azathioprine, methotrexate (MTX), and mycophenolate mofetil (MMF) have also been proposed as alternative agents. In the event of acute exacerbations, current guidelines advise considering a short course of systemic glucocorticosteroids. Recent advances in understanding the pathogenesis of CSU have facilitated the development of promising new therapies targeting novel molecular pathways, including receptors (FcεRI, C5aR, MRGPRX2), signaling molecules (BTK, SYK), and inflammatory mediators (IL-4, IL-17, IL-31) (16). These investigational therapies aim not only to alleviate symptoms but also to modify the natural course of the disease (disease-modifying treatments - DMTs) (18).

Moreover, recent research has increasingly focused on identifying patient-specific characteristics and biomarkers to support personalized treatment strategies in CSU aiming to move beyond the traditional trial-and-error approach. A major advancement in this effort is the stratification of CSU into distinct pathogenesis-based subtypes, guided by blood biomarkers and laboratory investigations. The early identification of predictors of response to SgAHs and OMA represents a clinically relevant objective. Several demographic and laboratory parameters have been explored as potential markers of treatment response. Among the most relevant demographic factors are age, sex, disease duration at diagnosis, disease severity, the presence of angioedema, inducible components, and comorbidities. Laboratory parameters include complete blood count, inflammatory markers, coagulation factors, and inflammatory molecules (such as cytokines), as well as immune system markers (such as ANA and anti-thyroid antibodies). While these studies offer valuable insights, findings have often been inconsistent, largely due to small sample sizes, methodological variability, and heterogeneous patient populations. Despite these limitations, ongoing efforts to identify reliable early predictors hold promises for optimizing CSU management. This review synthesizes the current evidence on markers associated with response to SgAHs and OMA, underscoring their potential to improve therapeutic decision-making and enhance patient outcomes.

**Figure 1:**
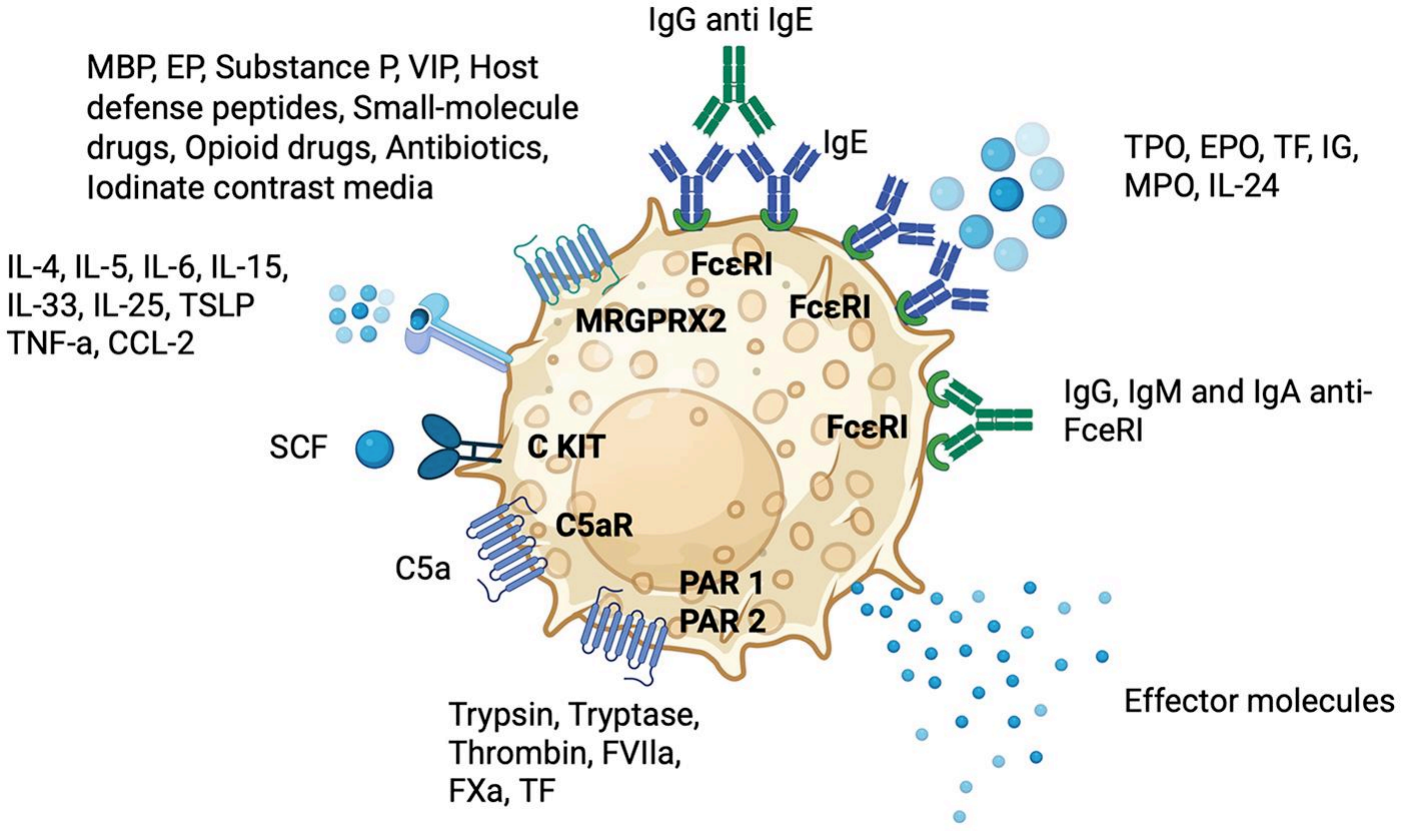
Pathogenic mechanisms underlying mast cell activation in CSU. C5aR, C5a receptor; CCL, C-C motif chemokine ligand; c-Kit, stem-cell factor receptor; CSU, chronic spontaneous urticaria; EPO, eosinophil peroxidase; FceRI, high-affinity IgE receptor; FVIla, activated factor VII; FXa, activated factor X; IL, interleukin; MBP, major basic protein; MC, mast cell; MRGPRX2, Mas-related G-protein-coupled receptor X2; PAR, protease-activated receptor; SCF, stem-cell factor; TF, tissue factor; TG, thyroglobulin; TNF, tumor necrosis factor; TPO, thyroid peroxidase; TSLP, thymic stromal lymphopoietin; VIP, vasoactive intestinal peptide.

A visual summury of different pathogenic mechanisms of CSU is presented in Figure 1.

## 2 Search methodology

A comprehensive literature search was carried out using PubMed and Google Scholar databases. The search terms included “chronic spontaneous urticaria” combined with “antihistamines”, “omalizumab”, “predictors”, “treatment response”, and “early response”. Articles published up to April 2025 were considered.

## 3 Antihistamines

SgAH1 are the first-line treatment for CSU reducing vascular permeability, edema, and sensory nerve stimulation, thereby alleviating urticaria symptoms (16). Several clinical and laboratory factors have been implicated in the reduced efficacy of sgAHs in patients with CSU, suggesting the presence of more active and complex disease phenotypes. The coexistence of inducible urticaria is a predictive factor of poor response to antihistamines, often necessitating earlier escalation to second-line therapies (19). This supports the notion that patients with mixed forms of urticaria represent a more difficult-to-treat subgroup from the outset of treatment. Among clinical markers, a high UAS7 score has emerged as the most robust indicator of disease severity and is consistently associated with poorer treatment response (19–22). From an immunoinflammatory perspective, elevated levels of C-reactive protein (CRP) and interleukin-6 (IL-6) reflect ongoing systemic inflammation frequently observed in non-responders (20, 23–25). Hematological parameters further support this inflammatory pattern. An increased neutrophil-to-lymphocyte ratio (NLR) indicates a proinflammatory immune imbalance (26). Reduced basophil and eosinophil counts suggest persistent cellular activation or tissue recruitment (20, 27, 28). Coagulation abnormalities also play a role in antihistamine resistance. Increased mean platelet volume (MPV), elevated D-dimer, and fibrinogen levels suggest coagulation cascade activation, potentially exacerbating inflammation, and vascular dysfunction (10, 20, 29, 30). An autoimmune involvement is suggested by positive ASST, BAT, and by the presence of autoantibodies such as anti-TPO, ANA, and rheumatoid factor (27, 31–33). Although ASST does not have a defined diagnostic value for CSU, its main relevance lies in its frequent co-segregation with TIIbaiCSU, as supported by recent evidence (34, 35). Elevated eosinophil cationic protein (ECP) and total IgE levels suggest the involvement of a TIaiCSU response, contributing to therapeutic resistance (30, 33). Atopic comorbidities, including asthma, allergic rhinitis, or atopic dermatitis, have also been associated with a lower probability of treatment response (36).

Conversely, high responsiveness to sgAH1 is associated with a shorter disease duration and low levels of inflammatory markers (e.g., CRP, D-dimer, ECP) and may indicate a less complex disease phenotype. These patients also often display lower disease activity scores (e.g., UAS7) (19, 20, 28, 30, 33). Interestingly, lower levels of diamine oxidase (DAO) have also been linked to better treatment outcomes, implicating altered histamine metabolism (37). Moreover, CSU patients, particularly those with histamine receptor 1 antagonist (H1RA) refractoriness, showed significant increases in serum platelet activating factor (PAF) levels and decreases in PAF acetylhydrolase (PAF-AH) (21). PAF is an endogenous, active phospholipid released from inflammatory cells, platelets, and endothelial cells, and is involved in the regulation of immune responses.

A summary of these findings is presented in Table 1.

**Table 1:** Positive and negative sgAHs response predictors.

Marker type	Negative predictors (Poor response to sgAHs)	Positive predictors (Good response to sgAHs)
Clinical markers	High UAS7 score, presence of inducible urticaria, long disease duration	Low disease activity, short disease duration
Inflammatory markers	Elevated CRP, IL-6	Low CRP, D-dimer, ECP, IL9
Hematologic markers	High NLR, low eosinophil and basophil counts, PAF	Normal or high eosinophil counts
Coagulation markers	Increased MPV, D-dimer, fibrinogen	Normal D-dimer, low fibrinogen
Immunological markers	High total IgE, high ECP	Low total IgE, low ECP
Autoimmune markers	Positive ASST, BAT, anti-TPO, ANA, RF	Negative ASST, ANA, RF
Histamine metabolism	–	Low DAO

## 4 Omalizumab

OMA is a recombinant humanized monoclonal antibody with a high safety profile, approved as the first biologic therapy for CSU patients unresponsive to sgAH1. Its mechanism of action is complex: it selectively binds to the Cε3 domain of immunoglobulin E (IgE), preventing its interaction with the high-affinity receptor FcεRI located on the membrane of mast cells, basophils, and antigen-presenting cells. This blockade leads to a progressive downregulation of FcεRI expression and ultimately reduces cellular activation, degranulation, and the release of histamine and other proinflammatory mediators (38). OMA represents a change in thinking in the treatment landscape, offering a mechanism-driven approach in a condition where traditional therapeutic options have often been inadequate. In clinical trials and real-world studies, approximately 40% of patients achieve complete remission of symptoms within 12 weeks of treatment, while 50%–70% exhibit partial responses. Nevertheless, a significant subgroup of patients—defined as late responders—require up to six months of therapy before clinical benefit is observed, and treatment interruption typically results in disease recurrence within a few months, although re-administration of OMA often restores disease control rapidly and effectively (39). It is estimated that approximately 20%–30% of patients do not respond to therapy with OMA. Predictive factors for poor response include several demographic and clinical features such as advanced age (36) and elevated body mass index (BMI) (40, 41). Other predictors include more severe disease activity, as reflected by higher UAS7 scores, longer disease duration, and more rapid relapse upon treatment discontinuation (36).

The coexistence of inducible urticaria (CIndU) in patients with CSU is generally considered a negative prognostic factor for response to OMA. Several real-world and retrospective studies have shown that patients with both CSU and CIndU—such as symptomatic dermographism, cold urticaria, or delayed pressure urticaria—tend to exhibit a slower, less complete, or absent clinical response to OMA compared to those with isolated CSU. Concomitant angioedema has also been associated with non-response in both groups.

Immunologically, several biomarkers have been associated with a reduced likelihood of response to OMA. These include the presence of antinuclear antibodies (ANA) (42). IgG or IgE antibodies against FcεRI and FcεRII, and comorbid autoimmune diseases (AIDs) such as Hashimoto's thyroiditis and systemic lupus erythematosus (36, 43, 44).

The potential of serum IgE levels as a predictive biomarker for omalizumab response in CSU has been the focus of numerous studies. One early investigation found that non-responders exhibited significantly lower baseline IgE levels, and their post-treatment IgE increases were also less pronounced. Notably, a two-fold or greater rise in IgE by week 4 of omalizumab therapy was identified as a strong indicator of treatment response (42). Supporting these findings, another two studies observed that individuals with low baseline IgE concentrations (0–15.2 IU/ml in Straesser cohort and 40–50 IU/ml in Marzano cohort) were less likely to respond to omalizumab (37, 45). Finally, a case series involving CSU patients with selective IgE deficiency reported minimal response to omalizumab (46). More recently, a meta-analysis involving 866 CSU patients reported significantly higher baseline total IgE levels in both complete and partial responders compared to non-responders, with mean differences of 56.5 IU/ml and 62.7 IU/ml, respectively, higher baseline total serum IgE predicts a better—and faster—clinical response responders (47). Nonetheless, a considerable proportion of patients with severe CSU and low IgE levels show a rapid response to omalizumab, indicating that IgE levels alone are not a definitive predictor of treatment outcome (48).

Basopenia and eosinopenia, which are considered surrogate markers of systemic immune activation, are associated with a poor response, as shown in a large study of over 1,600 CSU patients (28). Another marker, CD203c activity on basophils, was linked to reduced responsiveness, especially in patients with presumed autoimmune urticaria. Patients with lower CD203c activity showed better clinical outcomes after omalizumab treatment (49). A positive ASST or basophil histamine release assay (BHRA) has also been associated with delayed or reduced response (50).

Atopic comorbidities, including asthma, allergic rhinitis, or atopic dermatitis, have also been associated with a lower probability of treatment response (36). This is also relevant as they serve as a marker for the likelihood of type I CSU (51).

Conversely, several biomarkers have been positively correlated with omalizumab efficacy. Baseline basophilia has been shown to be associated with a favorable outcome (27), while high FcεRI expression on basophils—detectable via flow cytometry—has emerged as a robust predictor of early response, particularly in fast responders (52).

Notably, patients who lack basophil CD203c upregulating activity—which reflects the absence of circulating basophil-activating IgG autoantibodies—tend to exhibit faster and more sustained responses to OMA (28, 49, 53). Several cytokines and inflammatory markers have also been studied. Serum IL-31 levels decrease significantly following successful treatment, suggesting a role in the modulation of disease activity (54). Furthermore, despite the known association between elevated D-dimer and disease activity, this marker has not proven predictive of omalizumab response and is thought to reflect systemic vascular involvement or microthrombotic activity (27, 29, 36).

High levels of C-reactive protein (CRP) have been associated with acute, potentially post-infectious forms of CSU, which often show spontaneous remission (27).

Despite the overall efficacy of OMA, relapse after treatment discontinuation remains a frequent challenge, particularly in patients with initially high IgE levels or elevated FcεRI expression, both of which have been linked to shorter relapse-free survival (42) and in those with high baseline disease activity (55).

Interestingly, slow responders may possess cell-bound IgE or anti-FcεRI autoantibodies that interfere with the detection of FcεRI during flow cytometric analysis, potentially leading to an underestimation of receptor expression (50).

The presence of these immunological features highlights the heterogeneity of CSU and underscores the importance of identifying endotype-specific biomarkers to tailor therapy. Recent advances also support the use of composite immunological signatures—including basophil activation profile, FcεRI density, IL-31 levels, and total IgE—to refine therapeutic algorithms and stratify patients before treatment initiation (42, 56–58). A summary of these findings is presented in Table 2.

**Table 2:** Positive and negative omalizumab response predictors.

Category	Positive predictive factors (response)	Negative predictive factors (non-response)
Clinical	- Recent disease onset - Low disease activity - Absence of angioedema	- Advanced age - Elevated BMI - Severe disease (high UAS7) - Long disease duration - Presence of angioedema
Comorbidities	- Absence of atopic or autoimmune comorbidities	- Coexisting inducible urticaria (CIndU) - Asthma, allergic rhinitis, atopic dermatitis - Autoimmune diseases (e.g., Hashimoto's, SLE) - Hypertension
Serological biomarkers	- High total IgE - Decrease in IL-31 after treatment - Decline in D-dimer levels	- Low total IgE (<40–50 IU/ml) - Positive anti-TPO antibodies - Elevated CRP (in acute cases) - Elevated CRP (controversial) -Elevated ANA
Blood cell profile	- Basophilia	- Basopenia- Eosinopenia
Functional/skin tests	- Negative ASST - Negative BHRA - Negative BAT	- Positive ASST- Positive BHRA - Positive BAT (CD63↑) - Elevated CD203c
Basophil phenotype	- High FcεRI expression - Absence of CD203c upregulation	- Low FcεRI expression (may be artifact due to cell-bound IgE or anti-FcεRI antibodies)
Treatment course	- Rapid and sustained clinical response	- Slow response - Early relapse after treatment discontinuation

## 5 Cyclosporin

According to the most recent European urticaria guidelines (1), cyclosporin, with a dosage of 3.5–5 mg/kg per day, is reserved for patients who have not responded to high doses of SgAHs and omalizumab (16). A number of studies suggest that cyclosporine may be particularly effective in patients with Type IIb autoimmunity CSU (59). Individuals with a positive autologous serum skin test (ASST) have shown higher response rates to cyclosporine than ASST-negative patients, and ASST reactivity has been reported to diminish following treatment (60, 61). Elevated CRP levels have also been associated with improved response to cyclosporine (62, 63). Regarding IgE levels, cyclosporine responders tend to have lower concentrations than non-responders, suggesting a possible association between reduced IgE and improved treatment outcomes (64, 65). Furthermore, a positive basophil histamine release assay (BHRA) has also been associated with a better response to cyclosporine, as demonstrated in a larger cohort analysis involving 398 patients (66). In a systematic review evaluating predictors across 13 studies, the authors concluded that, despite variability in the strength of evidence, positive ASST, BHRA and BAT, elevated D-dimer, IL-2, IL-5 and TNF-α, and low serum IgE appear to be associated with greater likelihood of therapeutic benefit (67). More recently, features such as positive ASST, family history of CSU, elevated CRP and ESR, basopenia, eosinopenia, low IgE levels, and anti-TPO positivity were more frequently observed in patients responding exclusively to cyclosporine compared with omalizumab, although these associations were not statistically significant (68). Overall, biomarkers characteristic of type IIb autoimmune CSU, particularly ASST, BHRA or BAT positivity, low IgE, and elevated CRP, may provide useful indications of a more favorable response to cyclosporine therapy.

## 6 Others immunosuppressant drugs

Despite suggestions that immunomodulatory agents such as methotrexate, azathioprine, hydroxychloroquine, and dapsone may be more effective in patients with autoimmune features of CSU, particularly those with findings suggestive of type IIb autoimmune pathogenesis, the available evidence has not identified any reliable biomarkers that predict response to these therapies (59). Further research is therefore needed to clarify their clinical utility and to identify potential markers that could help guide their use.

## 7 Conclusion

The management of CSU remains a complex clinical challenge due to its heterogeneous pathophysiology and variable treatment responses. While sgAH1 represents the established first-line therapy, a considerable proportion of patients exhibit suboptimal or no response, necessitating early identification of refractory phenotypes. A growing body of evidence supports the role of multiple predictive markers including clinical features, inflammatory and hematologic profiles, coagulation parameters, immunological activity, and histamine metabolism in stratifying patients according to their likelihood of responding to antihistamines.

OMA has dramatically transformed the therapeutic landscape of CSU by targeting IgE-mediated pathways. Nevertheless, approximately one-third of patients experience delayed, partial, or absent responses to this treatment. Predictive biomarkers such as total IgE levels, FcεRI expression, CD203c basophil activity, IL-31, and D-dimer dynamics have emerged as promising tools to refine patient selection and therapeutic timing. Conversely, the presence of comorbid inducible urticaria, autoimmune markers, or persistent systemic inflammation may identify non-responders early in the clinical course. Together, these findings reinforce the concept that CSU encompasses multiple immunological endotypes, each with distinct biological behavior and treatment responsiveness. The incorporation of composite biomarker panels and validated clinical predictors into routine practice holds great potential to personalize CSU therapy, improve outcomes, and minimize unnecessary treatment delays. Among emerging therapies, dupilumab may display a biomarker profile similar to omalizumab, as both modulate IgE-related pathways, including the downregulation of FcεRI on mast cells and basophils. In contrast, remibrutinib, a selective BTK inhibitor, acts intracellularly on mast-cell signaling and is therefore expected to exert its efficacy largely independently of traditional CSU biomarkers. These mechanistic distinctions may shape the future landscape of predictive markers and personalized therapy. Future prospective studies are needed to validate these markers and establish algorithmic frameworks that integrate clinical, serological, and cellular indicators into decision-making pathways.
